# Detecting Endothelial Compromise Objectively through Dynamic EASIX scoring (DECODE) - predictive utility of EASIX and modified EASIX scores for endothelial complications post hematopoietic cell transplant

**DOI:** 10.3389/fonc.2026.1742467

**Published:** 2026-02-11

**Authors:** Saad Ghafoor, Kimberly Uchida, Jennifer McArthur, Yvonne Avent, Chia-Wei Hsu, Haitao Pan, Lama Elbahlawan

**Affiliations:** 1 Department of Pediatric Medicine, Division of Critical Care Medicine, St. Jude Children’s Research Hospital, Memphis, TN, United States; 2 Department of Pediatrics, Division of Pediatric Critical Care, University of Tennessee Health Science Center, Memphis, TN, United States; 3 Department of Pediatrics, Division of Pediatric Critical Care, University of Utah, Salt Lake City, UT, United States; 4 Department of Biostatistics, St. Jude Children’s Research Hospital, Memphis, TN, United States

**Keywords:** EASIX score, endothelial injury, endotheliopathy, hematopoietic cell transplantation, modified EASIX score, pediatric, sinusoidal obstruction syndrome, transplant-associated thrombotic microangiopathy

## Abstract

**Introduction:**

Endothelial injury is a major contributor to morbidity and mortality in pediatric patients undergoing hematopoietic cell transplantation (HCT). It appears as sinusoidal obstruction syndrome (SOS) or transplant-associated thrombotic microangiopathy (TMA), among other conditions. Composite indices like the Endothelial Activation and Stress Index (EASIX) and its modified version (m-EASIX) may serve as accessible biomarkers for early identification. However, their utility in pediatric populations is unestablished. We aimed to explore whether EASIX and m-EASIX can help identify endothelial complications in this setting.

**Methods:**

We conducted a prospective, single-center observational cohort study of 31 children and young adults undergoing HCT. Serial measurements of EASIX and m-EASIX scores, based on standard laboratory parameters, were collected at baseline and at multiple post-HCT time points (Days 0, 7, 14, 21, 28, and 100).

**Results:**

Within 100 days after HCT, SOS and/or TMA developed in six patients. At Day 21, EASIX and m-EASIX scores were significantly higher in children with endothelial complications than in controls. The m-EASIX score also showed predictive value at Day 14. Receiver operating characteristic analysis showed discrimination at Day 21 for both scores (AUCs of 0.807 for EASIX and 0.865 for m-EASIX). Changes from baseline to Day 21 further improved accuracy, with thresholds achieving high sensitivity for screening patients at increased risk of SOS and/or TMA. The Day 21 landmark is most relevant for identifying patients at risk of later-onset or persistent endothelial injury, which remains clinically significant.

**Conclusions:**

Our findings suggest that EASIX and m-EASIX may serve as practical and dynamic biomarkers for detecting endothelial injury in pediatric HCT recipients. The observation that Day 21 scores and their changes from baseline correlate with later complications highlights a potential window for risk stratification. However, these results should be interpreted cautiously, given the single-center design and limited sample size. Further research is needed to confirm whether these indices can reliably guide clinical decisions across diverse settings. Exploring their use in populations where reduced-intensity conditioning (RIC) and alternative donors are standard could provide important insights. Multicenter studies will be essential to validate these preliminary observations and refine biomarker-based strategies for post-HCT care.

## 1 Introduction

Endothelial injury is a key contributor to morbidity and mortality after pediatric hematopoietic cell transplantation (HCT). The endothelium is a dynamic interface between circulating blood and tissue. It plays a crucial role in regulating vascular tone, coagulation, and immune function, and it maintains barrier integrity. In the post-HCT setting, the endothelium is often disrupted by cytotoxic conditioning regimens, immunosuppressive agents, infections, and immune reconstitution, resulting in endothelial dysfunction syndromes, such as sinusoidal occlusive syndrome (SOS) and transplant-associated thrombotic microangiopathy (TMA) (1–3).

The underlying mechanisms of endothelial injury include pro-inflammatory cytokine activation, complement system dysregulation, and direct endothelial cytotoxicity. SOS and TMA represent severe, persistent forms of endothelial injury: SOS is characterized by hepatic sinusoidal blockage, and TMA is characterized by widespread microvascular thrombosis. Both syndromes lead to significant organ damage and adverse outcomes (4, 5). Pediatric patients are particularly vulnerable due to their smaller vascular volumes, immature immune systems, and limited physiological reserves. Endothelial dysfunction in this population increases the need for intensive care, extends hospitalizations, and contributes to long-term organ complications (6–8).

Despite its clinical significance, identifying evolving endothelial injury early remains challenging. Traditional diagnostic methods, which rely on clinical symptoms and isolated laboratory findings, often lack adequate sensitivity and specificity. Invasive procedures, such as biopsies and advanced imaging, are seldom feasible in children. This diagnosis underscores the need for accessible, reliable biomarkers that can identify endothelial stress before irreversible damage occurs. Composite scores derived from routine laboratory values offer a pragmatic solution. The Endothelial Activation and Stress Index (EASIX), calculated from lactate dehydrogenase (LDH), creatinine, and platelet count, reflects systemic endothelial stress and organ dysfunction and can be monitored serially at the bedside (9).

The ease of use and reproducibility of EASIX are particularly beneficial in pediatric settings, where frequent assessments are required (10). Recent evidence suggests that a modified EASIX (m-EASIX) score, which substitutes C-reactive protein (CRP) for Creatinine, enhances the predictive value for severe, persistent endothelial complications, such as SOS and TMA (11). The EASIX and m-EASIX scores are strongly correlated with clinical outcomes, including nonrelapse mortality, intensive care unit (ICU) admission, and organ failure. Significantly, they facilitate risk stratification at critical time points before transplantation, during conditioning, and in the early post-HCT phase. This enables clinicians to identify patients at increased risk before their overt clinical decline (12).

Whether EASIX and m-EASIX scores can predict endothelial injury in pediatric HCT recipients remains insufficiently characterized. To address this gap, we prospectively studied the relations between the EASIX and m-EASIX scores and the development of SOS and/or TMA in children undergoing HCT. We hypothesized that elevated EASIX and m-EASIX values at specific post-HCT time points would be associated with the onset of these complications. Additionally, we aimed to identify clinically relevant thresholds for both scoring systems to facilitate early prediction of endothelial injury in routine practice.

## 2 Materials and methods

### 2.1 Study population

This single-center, prospective, observational cohort study was conducted from 11/28/2022 to 1/18/2024. Eligibility criteria included patients aged 22 years or younger who weighed at least 10 kg. Individuals were excluded if they exceeded the age or weight limits or if consent was not provided. Study follow-up extended through Day 100 post-HCT for endpoint ascertainment. There were 28 allogeneic and three autologous transplants. The study protocol was approved by the Institutional Review Board at St. Jude Children’s Research Hospital, and written informed consent was obtained from all participants or their guardians, as appropriate.

### 2.2 Data collection

Comprehensive data were collected for each participant, including demographic variables (i.e., age, sex, race, and ethnicity) and details about their underlying malignancy, HCT type, donor characteristics, conditioning regimen, and survival outcomes. Clinical information on ICU admission, the use of invasive mechanical ventilation, the administration of vasopressors or inotropic agents (e.g., norepinephrine, epinephrine, dobutamine, phenylephrine, vasopressin, dopamine, and milrinone), and renal replacement therapy was also documented. Blood samples were taken at baseline (day of HCT, Day 0) and on Days 7, 14, 21, 28, and 100 after the transplantation. At each time point, laboratory parameters, including serum creatinine, LDH, platelet count, and CRP were measured and recorded.

### 2.3 Definitions

Endothelial complications were defined by the occurrence of TMA and/or SOS. The earliest date of diagnosis was used as the index date for analysis. A diagnosis of TMA was made based on a documented clinical evaluation and management by the treating physician, histologic confirmation, or fulfillment of the Modified Jodele Criteria (13). These criteria include laboratory and clinical markers, such as elevated LDH, proteinuria, and refractory cytopenias. Similarly, SOS was diagnosed according to the pediatric criteria determined by the European Society for Blood and Marrow Transplantation (5) or based on a clinical diagnosis documented in the medical records and managed accordingly. The EASIX score was calculated using the following formula: serum LDH level (U/L) × creatinine level (mg/dL)/platelet count (10^9^/L) (14). The m-EASIX score was calculated based on the following formula: serum LDH level (U/L) × CRP (mg/dL)/platelet count (10^9^/L) (15). The values from both scores were normalized using log_2_ transformation to reduce skewness, as reported in the literature.

### 2.4 Statistical analysis

Descriptive statistics were used to summarize patient characteristics. Continuous variables were reported as medians with interquartile ranges (IQR) and were compared using the Wilcoxon signed-rank test. Categorical variables were compared using the Chi-square test or Fisher’s exact test if fewer than five observations were included.

The EASIX and m-EASIX scores were log_2_-transformed and analyzed at Days 0, 7, 14, 21, 28, and 100. These predefined time points were selected to capture peri-transplant endothelial dynamics and potential predictive windows for post-HCT complications. Due to the limited number of individual events, we used a composite endpoint of SOS/TMA to increase statistical power. Separate analyses for each complication were not feasible, given the small sample size.

Univariate logistic regression was used to identify factors associated with SOS and/or TMA. A small number of events precluded reliable multivariable modeling, and larger studies are needed for adjusted analyses. Results were reported as odds ratios (OR) and 95% confidence intervals (CI). At landmark time points (Days 7, 14, and 21), predictive performance was evaluated using receiver operating characteristic (ROC) analysis, with areas under the curve (AUCs) and optimal cutoffs determined by the Youden index, closest-to-(0,1) method, and ≥90% sensitivity criterion (16). Dynamic changes (ΔEASIX and Δm-EASIX) were defined as the difference between each post-HCT value and the corresponding baseline value, and their predictive utility was similarly evaluated using ROC analysis. All statistical tests were two-sided, with p <0.05 considered significant. Analyses were performed using SAS version 9.4.

## 3 Results

A total of 31 children participated in this prospective study. Within the first 100 days after HCT, six (19%) children experienced either TMA or SOS. The median age of the group was 11 years, and males comprised 58% of the cohort (**Table 1**). Patients with or without SOS and/or TMA had similar median ages (10 vs 12.5 years, p = 0.82) and sex distributions (50% vs 60% male, p = 0.68). Most patients received allogeneic HCT (90%), mainly with reduced-intensity conditioning (68%) and a mismatched related donor (MMRD) (64%). The main reason for HCT was malignant disease (77%), most often acute myeloblastic leukemia (AML).

**Table 1:** Characteristics of the cohort.

Characteristic	All cohort (N = 31)	SOS/TMA (*n* = 6)	No SOS/TMA (*n* = 25)	P-value
Age (years)	11 (2–22)	10 (2–18)	12.5 (2–22)	0.82
Sex				0.67
*Female*	13 (41.94)	3 (50.00)	10 (40.00)	
*Male*	18 (58.06)	3 (50.00)	15 (60.00)	
Ethnicity				0.34
*Hispanic*	12 (38.71)	4 (66.67)	8 (32.00)	
*Non-Hispanic*	18 (58.06)	2 (33.33)	16 (64.00)	
*Unknown*	1 (3.23)	0 (0.00)	1 (4.00)	
Race (%)				0.64
*White*	19 (61.29)	3 (50.00)	16 (64.00)	
*Black*	5 (16.13)	1 (16.67)	4 (16.00)	
*Unknown*	4 (12.90)	2 (33.33)	2 (8.00)	
*American Indian/Alaskan Native*	1 (3.23)	0 (0.00)	1 (4.00)	
*Mixed*	1 (3.23)	0 (0.00)	1 (4.00)	
*Native Hawaiian/Other*	1 (3.23)	0 (0.00)	1 (4.00)	
Conditioning regimen				0.32
*Myeloablative*	9 (30.00)	3 (50.00)	6 (25.00)	
*Reduced intensity*	21 (68.00)	3 (50.00)	18 (75.00)	
Donor type				0.49
*Mismatched-related*	18 (64.29)	3 (50.00)	15 (68.18)	
*Matched sibling*	3 (10.71)	1 (16.67)	2 (9.09)	
*Matched-unrelated*	7 (25.00)	2 (33.33)	5 (22.73)	
Type of HCT				1
*Allogeneic*	28 (90.32)	6 (100.00)	22 (88.00)	
*Autologous*	3 (9.68)	0 (0.00)	3 (12.00)	
HCT indication				0.70
*ALL*	6 (19.35)	2 (33.33)	4 (16.00)	
*AML*	12 (38.71)	2 (33.33)	10 (40.00)	
*CML*	2 (6.45)	1 (16.67)	1 (4.00)	
*Nonmalignant hematologic disorder*	7 (22.58)	1 (16.67)	6 (24.00)	
*Lymphoma*	1 (3.23)	0 (0.00)	1 (4.00)	
*Solid tumor*	3 (9.68)	0 (0.00)	3 (12.00)	
ICU Admission				0.11
*No*	24 (77.42)	3 (50.00)	21 (84.00)	
*Yes*	7 (22.58)	3 (50.00)	4 (16.00)	

ALL, acute lymphoblastic leukemia; AML, acute myeloblastic leukemia; AUTO, autologous; CML, chronic myelogenous leukemia; HCT, hematopoietic cell transplantation; ICU, intensive care; SOS, sinusoidal obstruction syndrome; TMA, thrombotic microangiopathy.

The rate of use of reduced-intensity conditioning (RIC) regimens is 68% (21 of 31). The rate of use of Busulfan is 19% (6 of 31). The median day of diagnosis for SOS/TMA was Day 21 (Day 10–94). Four events occurred on or before Day 21, and two occurred after (Days 63 and 94). Of the four SOS cases, three were severe, and one was moderate.

Our institutional practice for a significant portion of allogeneic transplants, often driven by concerns about organ toxicity, prior receipt of intensive therapy, or the underlying diagnoses, is to use a RIC regimen. This represents a specific population. None received TBI.

Notably, seven (22%) patients underwent HCT for nonmalignant hematologic conditions. Seven (22%) patients needed ICU admission. Among the six patients who experienced SOS and/or TMA, two had isolated TMA, one had isolated SOS, and three had both conditions concurrently (SOS/TMA). All patients with SOS received defibrotide, and four of five patients with TMA received eculizumab. Of those who experienced SOS/TMA, two required invasive mechanical ventilation, two needed vasopressors, and two underwent renal replacement therapy. Three patients in the cohort died, with two deaths occurring more than 100 days after HCT.

### 3.1 EASIX and SOS and/or TMA

**Figure 1:**
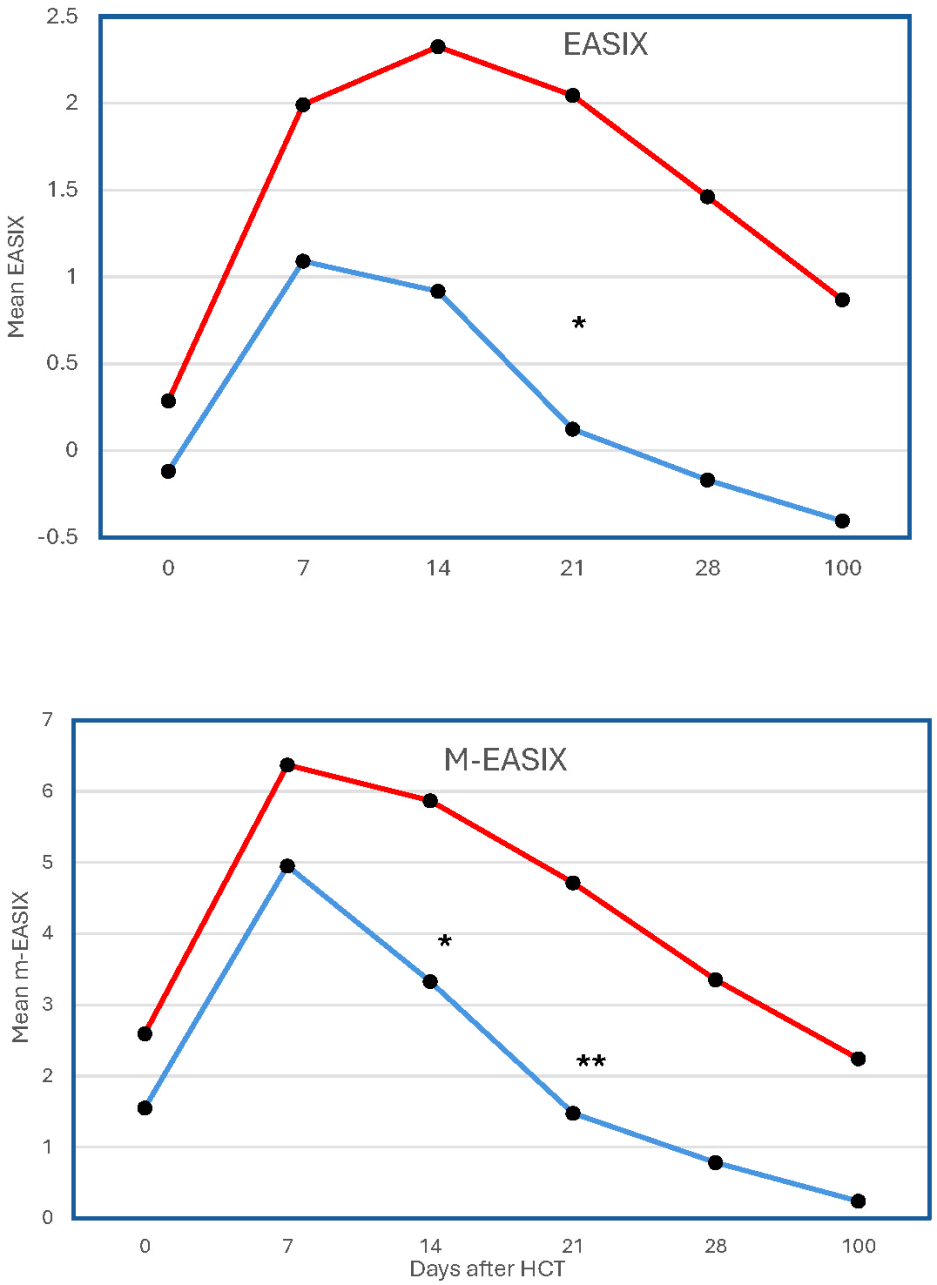
Temporal changes in the mean EASIX and m-EASIX score after HCT are depicted at Days 0, 7, 14, 21, 28, and 100. The red plot shows score trends in pediatric patients who experienced SOS and/or TMA, and the blue plot shows trends in those who did not. *p <0.05; **p <0.01.

On the day of transplantation, EASIX scores did not differ significantly between children who later developed TMA and/or SOS and those who did not (**Table 2**). However, by Day 21 post-HCT, patients in whom SOS/TMA developed had significantly higher median EASIX scores (2.27, IQR –0.35-3.91) compared to those in whom these complications did not develop (–0.44, IQR –1.72-3.19; p = 0.01) (**Table 3**). Furthermore, we observed higher median EASIX scores at Day 28 in children with SOS/TMA (1.55, IQR –0.70-4.61) than in those without (0.58, IQR –1.92-4.25; p=0.05). Both groups showed an increase in EASIX scores by Day 7; however, EASIX scores continued to rise only in those in whom SOS/TMA developed, with scores peaking at Day 14 (**Figure 1**). Across all time points, the mean EASIX scores were higher in the SOS/TMA group compared to controls, with statistically significant differences on Days 21 (p=0.01) and 28 (p=0.05).

**Table 2:** Association of the EASIX score with the development of SOS and/or TMA after pediatric HCT.

Day post-HCT	EASIX (log2)			m-EASIX (log2)		
	SOS/TMA^1^	No SOS/TMA^1^	P-value	SOS/TMA	No SOS/TMA	P-value
0	0.35 (–3.10-3.78)	–0.04 (–4.06-2.99)	0.68	3.23 (–1.08-5.03)	0.54 (–3.40-6.99)	0.36
7	1.98 (0.21-3.82)	1.27 (–1.32-2.81)	0.17	5.92 (4.49-8.85)	5.05 (0.50-7.40)	0.14
14	2.69 (–0.19-4.54)	0.94 (–1.94-4.08)	0.23	5.81 (3.52-9.63)	3.69 (–0.56-6.17)	0.01
21	2.27 (–0.35-3.91)	–0.44 (–1.72-3.19)	0.01	4.73 (2.39-7.54)	1.31 (–1.15-6.24)	<0.0001
28	1.55 (–0.70-4.61)	–0.58 (–1.92-4.25)	0.05	2.66 (–1.41-7.14)	0.55 (–1.57-5.29)	0.07
100	0.40 (–2.08-3.84)	–0.51 (–1.90-3.19)	0.45	–1.38 (–1.76-9.86)	–0.72 (–2.14-7.50)	1.00

Day post-HCT	EASIX (log2)			m-EASIX (log2)		
	SOS/TMA^1^	No SOS/TMA^1^	P-value	SOS/TMA	No SOS/TMA	P-value
0	1.27 (49.00-13.72)	0.97 (0.06-7.97)	0.68	10.56 (0.47-32.70)	1.45 (0.09-126.84)	0.36
7	4.32 (49.00-14.13)	2.41 (0.40-7.01)	0.17	65.06 (22.52-460.02)	33.07 (1.41-168.41)	0.14
14	10.94 (49.00-23.34)	1.92 (0.26-16.97)	0.23	58.60 (11.48-791.49)	12.90 (0.68-72.20)	0.01
21	5.10 (49.00-14.99)	0.74 (0.30-9.13)	0.01	26.57 (5.23-186.63)	2.49 (0.45-75.43)	0.00
28	2.93 (49.00-24.46)	0.67 (0.26-19.01)	0.05	6.30 (0.38-141.30)	1.49 (0.34-39.20)	0.07
100	1.32 (49.00-14.36)	0.70 (0.27-9.15)	0.45	0.38 (0.30-929.95)	0.61 (0.23-180.65)	1.00

^1^Values presented are the medians of log_2_ EASIX scores, with the ranges in parentheses.

EASIX, Endothelial Activation and Stress Index; HCT, hematopoietic cell transplant; SOS, sinusoidal obstruction syndrome; TMA, thrombotic microangiopathy.

**Table 3:** Univariate analysis of log_2_-EASIX.

Day 21	Univariate analysis of SOS/TMA		
Parameter	Category	OR (95% CI)	P-value
EASIX		2.12 (1.07-4.22)	0.03
Age at HCT		0.97 (0.83-1.13)	0.66
Conditioning regimen (ref=MAC)	RIC	0.24 (0.03-1.63)	0.14
Donor type (ref=MMRD)	MSD	2.33 (0.16-34.89)	0.54
	MUD	1.87 (0.24-14.65)	0.55

CI, confidence interval; HCT, hematopoietic cell transplantation; MAC, myeloablative conditioning; MMRD, mismatched-related donor; MSD, matched-sibling donor; MUD, matched-unrelated donor; OR odds risk; RIC, reduced-intensity conditioning; SOS, sinusoidal obstruction syndrome; TMA, thrombotic microangiopathy.

### 3.2 Modified EASIX and SOS/TMA

At baseline, there was no difference in m-EASIX scores between children who experienced SOS/TMA and those who did not (**Table 4**), thus mirroring the findings for EASIX scores. However, children in whom SOS/TMA developed showed significantly higher m-EASIX scores on Days 14 and 21 post-HCT than those without the complications. On Day 14, the median m-EASIX score was 5.81 (IQR 3.52–9.63) in the SOS/TMA group versus 3.69 (IQR –0.56-6.17; p = 0.01) in the non-SOS/TMA group. Similarly, on Day 21, the median score was 4.73 (IQR 2.39-7.54) compared to 1.31 (IQR –1.15-6.24; p <0.0001). All participants experienced a peak in their mean m-EASIX scores at Day 7 post-HCT, but scores remained consistently higher in the SOS/TMA group than in those without these complications (**Figure 1**).

**Table 4:** Univariate analysis of log_2_ m-EASIX.

Univariate analysis of SOS/TMA			
Parameter	Category	OR (95% CI)	P-value
Day 21			
m-EASIX		1.84 (1.09-3.11)	0.02
Age at HCT		0.97 (0.83-1.13)	0.66
Conditioning regimen (ref=MAC)	RIC	0.24 (0.03-1.63)	0.14
Donor type (ref=MMRD)	MSD	2.33 (0.16-34.89)	0.54
	MUD	1.87 (0.24-14.65)	0.55
Day 28			
m-EASIX		1.63 (0.99-2.68)	0.05
Age at HCT		0.96 (0.82-1.11)	0.56
Conditioning regimen (ref=MAC)	RIC	0.33 (0.05-2.13)	0.25
Donor type (ref=MMRD)	MSD	1.63 (0.11-22.98)	0.72
	MUD	1.30 (0.18-9.47)	0.80

CI, confidence interval; HCT, hematopoietic cell transplant; MAC, myeloablative conditioning; MMRD, mismatched-related donor; MSD, matched-sibling donor; MUD, matched-unrelated donor; OR, odds risk; RIC, reduced-intensity conditioning; SOS, sinusoidal obstruction syndrome; TMA, thrombotic microangiopathy; MAC, myeloablative conditioning.

### 3.3 Predictive performance of static EASIX and m-EASIX scores

To evaluate the predictive accuracy of EASIX and m-EASIX scores, we performed a landmark analysis. At each predetermined prediction time point (Days 7, 14, and 21), we assessed biomarker performance only in patients who remained free of events and at risk for future complications. Accordingly, the at-risk populations included 31 patients (6 future cases, 25 controls) at Day 7, 28 patients (3 future cases, 25 controls) at Day 14, and 27 patients (2 future cases, 25 controls) at Day 21.

We performed ROC analysis to evaluate the discriminative ability of single-time-point (static) EASIX and m-EASIX scores to distinguish patients who subsequently experienced SOS and/or TMA from those who did not. The AUC values and their 95% CIs, which were calculated via bootstrap resampling to account for sample-size limitations, are presented in **Table 5**. Both scores demonstrated the strongest predictive ability at Day 21. The EASIX achieved its peak discrimination at this time point (AUC 0.807, 95% CI: 0.520-1.000), and the m-EASIX also performed well (AUC 0.865, 95% CI: 0.591-1.000). Predictive accuracy at earlier time points (Days 7 and 14) was moderate for both scores.

**Table 5:** Predictive performance of static EASIX and m-EASIX scores.

Predictor	Landmark timepoint (day)	At-risk set (cases/controls)	AUC	Bootstrap 95% CI
EASIX	7	6/25	0.682	0.381-0.954
	14	3/25	0.674	0.453-0.885
	21	2/25	0.807	0.520-1.000
m-EASIX	7	6/25	0.686	0.377-0.908
	14	3/25	0.684	0.427-0.933
	21	2/25	0.865	0.591-1.000
Δ EASIX	D21-D0	2/25	0.761	0.376-1.000
Δ m-EASIX	D21-D0	2/25	0.764	0.379-1.000

AUC, area under the curve; CI, confidence interval.

**Figure 2:**
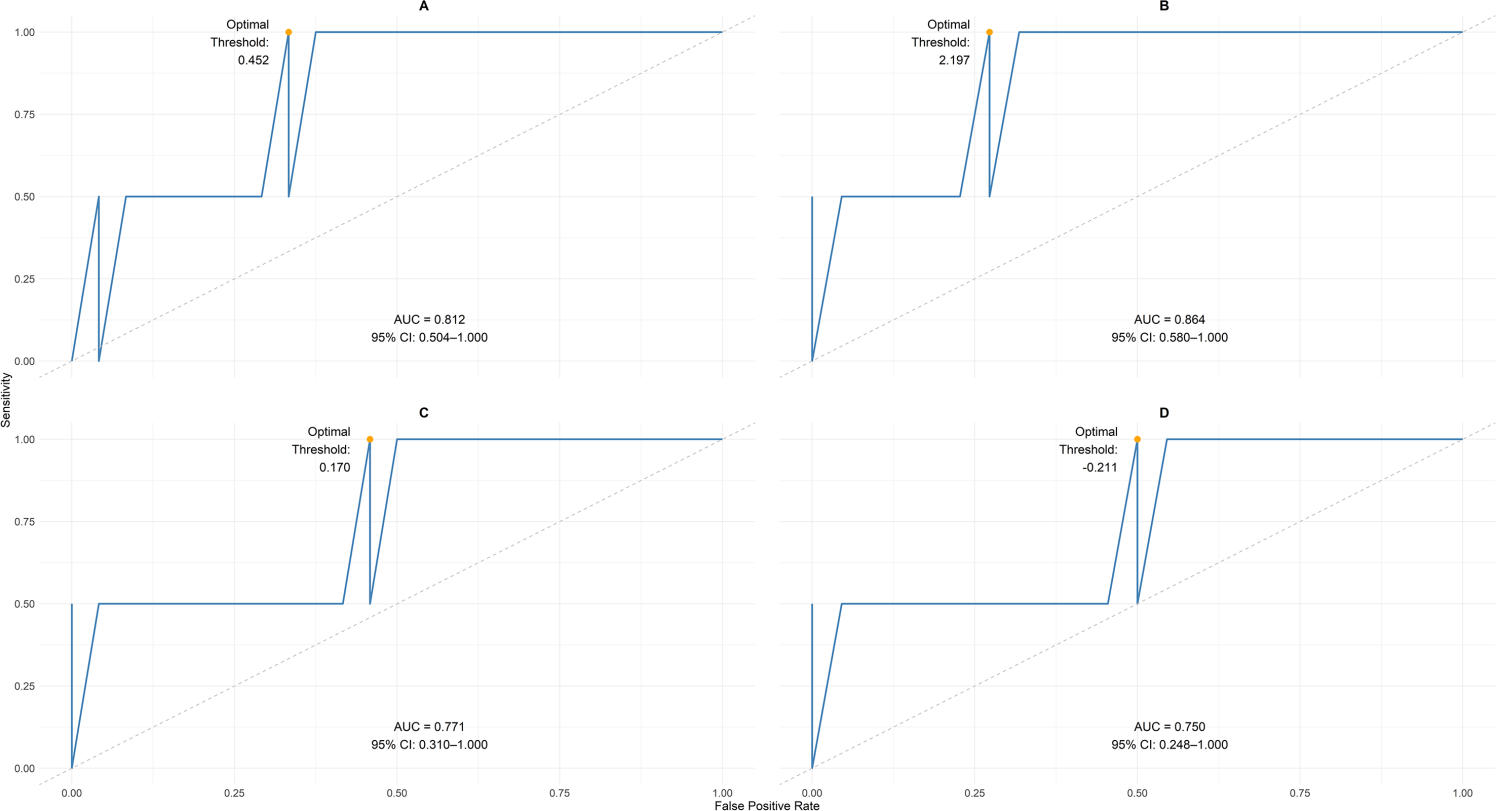
**(A)** The ROC curved derived from the statistic EASIX scores shows the predictive performance of the log2(EASIX) score on Day 21. The model achieved an AUC of 0.812 (95% CI: 0.504-1.000), indicating good discrimination. The optimal threshold (0.542) represents the best balance between sensitivity and specificity. **(B)** The ROC curved derived from the statistic modified-EASIX scores shows the predictive performance of the log2(m-EASIX) score on Day 21 post-HCT. The model achieved an AUC of 0.864 (95% CI, 0.580-1.00), indicating good discrimination. The optimal threshold (2.197) represents the best balance between sensitivity and specificity. **(C)** The ROC curved derived from dynamic EASIX scores shows the predictive performance of the log2(EASIX) score from baseline (Day 0) through Day 21 post-HCT. The model achieved an AUC of 0.771 (95%CI, 0.310-1.00), indicating good discrimination. The optimal threshold (0.170) represents the best balance between sensitivity and specificity. **(D)** The ROC curved derived from the dynamic m-EASIX scores shows the predictive performance of the log2(m-EASIX) score from baseline (Day 0) through day 21 post-HCT. The model achieved an AUC of 0.750 (95% CI, 0.248-1.00), indicating good discrimination. The optimal threshold (-0.211) represents the best balance between sensitivity and specificity.

For clinical application, optimal cut-off points for EASIX and m-EASIX on Day 21 were identified using three methods: the Youden index, the point closest to (0,1) on the ROC curve, and a high-sensitivity (≥90%) criterion. Notably, all three methods identified the same threshold for each score, indicating robust cut-off points. For EASIX, the optimal threshold was 0.452 (sensitivity 1.00, specificity 0.67) (**Figure 2A**); for m-EASIX, the threshold was 2.197 (sensitivity 1.00, specificity 0.73) (**Figure 2B**).

### 3.4 Dynamic changes in EASIX and m-EASIX

We investigated whether the fold-change in biomarker levels (Δlog_2_) over time provided greater predictive accuracy than single-timepoint measurements. The AUC values for predicting future endothelial complications based on changes in EASIX and m-EASIX scores from baseline (Day 0) are summarized in **Table 6**. The fold-change from Day 0 to Day 21 (ΔD21 –D0) yielded the highest discrimination for both biomarkers. An increase in EASIX from baseline to Day 21 (ΔEASIX D21 – D0) was a strong predictor, with an AUC of 0.761; the corresponding rise in m-EASIX (Δm-EASIX D21 – D0) was comparable (AUC 0.764). For the most predictive dynamic measure (ΔD21 – D0), the Youden index, the point closest to (0,1) on the ROC curve, and the high-sensitivity method identified the same optimal threshold for each composite score. For ΔEASIX (D21 – D0), the threshold was 0.170 (sensitivity 1.00, specificity 0.54) (**Figure 2C**); for Δm-EASIX (D21 – D0), the threshold was –0.211 (sensitivity 1.00, specificity 0.50) (**Figure 2D**).

**Table 6:** Predictive performance of dynamic EASIX and m-EASIX changes from baseline.

Predictor	Interval	Prediction	Landmark AUC	Bootstrap 95% CI
Δ EASIX	D7–D0	D7	0.627	0.381-0.893
	D14–D0	D14	0.707	0.469-0.960
	D21–D0	D21	0.761	0.376-1.000
Δ m-EASIX	D7–D0	D7	0.578	0.422-0.759
	D14–D0	D14	0.748	0.385-1.000
	D21–D0	D21	0.764	0.379-1.000

AUC, area under the curve; CI, confidence interval; D, days after hematopoietic cell transplantation.

## 4 Discussion

This prospective study demonstrates that both EASIX and m-EASIX scores may serve as valuable indicators of endothelial stress and help identify patients at risk for sinusoidal obstruction syndrome (SOS) and transplant-associated thrombotic microangiopathy (TMA) after hematopoietic cell transplantation (HCT) in a single-center cohort. This work contributes to the expanding literature on endothelial biomarkers in pediatric HCT by prospectively evaluating these indices and introducing the modified EASIX (m-EASIX) score in this population. To date, this is the first study to examine m-EASIX’s potential to predict SOS and TMA in children. Incorporating C-reactive protein (CRP) as a marker of inflammation, m-EASIX demonstrated encouraging discriminative performance (AUC 0.865 at Day 21) and slightly outperformed the classic EASIX score, possibly reflecting its ability to capture inflammatory endothelial stress. In addition to single-time-point measurements, dynamic changes in EASIX and m-EASIX relative to baseline also showed promising predictive value at Day 21. Notably, m-EASIX was elevated as early as Day 14, suggesting it may provide an earlier signal of endothelial injury than EASIX. These preliminary findings support further investigation into the utility of m-EASIX for risk stratification and monitoring in pediatric HCT. Given that SOS and TMA are biologically distinct entities, differences in biomarker kinetics are plausible. Low event rates necessitated the use of a composite endpoint, and larger studies are required to clarify whether these scores behave differently in SOS versus TMA.

These observations align with prior research. Muratore et al. reported that, in a cohort of 167 children after allogeneic HCT, the EASIX score at Day 7 was an independent predictor of SOS and nonrelapse mortality (17). In a multicenter study of 150 adults undergoing allogeneic HCT with myeloablative conditioning, circulating endothelial cell counts greater than 17/ml were associated with increased SOS risk (18). Baseline EASIX scores did not differ between groups, but elevated scores at engraftment correlated with SOS development (odds ratio 1.06, 95% CI: 1.03–1.11, p = 0.001). No relationship was observed between circulating endothelial cell levels and EASIX scores, suggesting that each has a marker for independent predictive value. Jiang et al. found that higher EASIX values on the day of transplant were associated with increased SOS/VOD incidence in both training and validation cohorts of adult patients, with odds ratios per log2 increase of 1.31 (95% CI: 1.08–1.59, p = 0.0067) and 1.57 (95% CI: 1.26–2.01, p = 0.0001), respectively (19). Additionally, Luft et al. noted that higher pretransplant EASIX scores correlated with increased TMA incidence and worse outcomes in univariate analysis, though these associations did not persist in multivariate models (20). Collectively, these studies and the present findings highlight the potential role of EASIX-based biomarkers in identifying patients at risk for serious endothelial complications following HCT. However, variability in timing and predictive strength across studies underscores the need for further research to define optimal assessment windows.

A notable observation was the lack of significant differences in EASIX and m-EASIX scores at baseline, suggesting similar initial endothelial stress across patients. Over time, however, persistent elevation of these scores distinguished those who developed complications, indicating that failure to resolve early endothelial injury may be more predictive than the initial insult. The data also suggest that m-EASIX, which substitutes CRP for creatinine, may offer modestly improved accuracy at Day 21, likely due to its inclusion of an inflammatory marker. Both single-point and dynamic changes at Day 21 yielded thresholds with high sensitivity, which could help identify patients requiring closer monitoring or early intervention.

In clinical practice, incorporating EASIX and m-EASIX scores into routine post-HCT monitoring appears feasible and cost-effective, as these indices rely on standard laboratory tests. Scores exceeding established thresholds by Day 21 should prompt clinicians to consider proactive measures, such as optimizing fluid balance, adjusting immunosuppression, enhancing infection surveillance, or initiating targeted therapies, including defibrotide or complement inhibitors. These biomarkers may also serve as objective tools for risk stratification in clinical trials evaluating preventive or preemptive strategies. We acknowledge that the Day 21 landmark is most relevant for identifying patients at risk of later-onset or persistent endothelial injury, a clinically significant concern.

While this study provides novel insights, several limitations warrant caution. The small number of events and single-center design limit generalizability, and the proposed thresholds should be considered exploratory. The heterogeneous cohort, which included both malignant and non-malignant indications and varied conditioning regimens, may also influence applicability to other settings. However, our findings may be especially relevant in populations where RIC and alternative donors are commonly used. Future multicenter studies with larger, more uniform populations are needed to validate these findings and explore associations with other post-HCT complications, such as graft-versus-host disease.

## 5 Conclusion

In summary, EASIX and m-EASIX scores show promise as practical, dynamic biomarkers for early detection of endothelial stress in pediatric HCT recipients. Identification of a potential predictive window at Day 21 and establishment of preliminary thresholds may support a more personalized approach to post-transplant care. Early recognition and intervention could help reduce ICU admissions and improve outcomes, but further validation is essential before these strategies can be widely implemented.

## Data Availability

The raw data supporting the conclusions of this article will be made available by the authors, without undue reservation.
